# A study of arterial stiffness in turner syndrome patients using cardio-ankle vascular index

**DOI:** 10.1186/1687-9856-2015-S1-P82

**Published:** 2015-04-28

**Authors:** Hae Woon Jung, Shin Mi Kim, Hwa Young Kim, Young Ah Lee, Sei Won Yang, Choong Ho Shin

**Affiliations:** 1 Department of Pediatrics, Seoul National University College of Medicine, Seoul, South Korea; 2 Seoul Red Cross Hospital, Seoul, South Korea

## 

A large proportion of the increased mortality in Turner syndrome (TS) is related to cardiovascular complications. Increased arterial stiffness may be an important predictor related to cardiovascular complications in TS patients. A novel method of evaluating arterial stiffness, which is relatively independent of changes in blood pressure (BP), is the cardio-ankle vascular index (CAVI). The aim of this study was to compare arterial stiffness using CAVI between TS patients and healthy controls and to evaluate for possible factors affecting arterial stiffness within the patient group. Known TS patients (n=24) with confirmed karyotypes were recruited from the outpatient clinic of Seoul National University Children’s Hospital between August, 2010 and June, 2013. Patients with type 2 diabetes and/or hypertension requiring medication were excluded. There were 5 patients with one or more combined congenital heart anomalies (aortic coarctation (n=2), bicuspid aortic valve (n=2), aortic stenosis (n=3). Anthropometric data, fasting blood lab and measurements of CAVI and pulse wave velocity were collected. A healthy control group (n=23) matched for age and body mass index (BMI) were recruited for comparison. The mean age and BMI of the TS patients were 27.0 years and 22.8 kg/m^2^ respectively while that of the control were 28.2 years and 22.04 kg/m^2^. CAVI was significantly higher in the TS patients compared to controls (6.05 vs. 6.65, P < 0.001), while there was no significant difference in pulse wave velocity. Univariate analysis for factors affecting CAVI within the TS patient group showed that CAVI was associated with waist circumference (P = 0.04) and systolic BP (P = 0.045). There were no significant factors related to CAVI using multivariate regression analysis including age, systolic BP, waist circumference, HOMA-IR and presence of cardiac anomalies. TS patients showed an increased arterial stiffness compared to age- and BMI-matched controls using CAVI measurement. Further prospective studies in larger TS patient group are mandatory in order to find significant factors related to increased arterial stiffness.

